# Structure and Dynamics of Amyloid-β Segmental Polymorphisms

**DOI:** 10.1371/journal.pone.0041479

**Published:** 2012-07-24

**Authors:** Workalemahu M. Berhanu, Ulrich H. E. Hansmann

**Affiliations:** Department of Chemistry and Biochemistry, University of Oklahoma, Norman, Oklahoma, United States of America; University of Akron, United States of America

## Abstract

It is believed that amyloid-beta (Aβ) aggregates play a role in the pathogenesis of Alzheimer’s disease. Aβ molecules form β-sheet structures with multiple interaction sites. This polymorphism gives rise to differences in morphology, physico-chemical property and level of cellular toxicity. We have investigated the conformational stability of various segmental polymorphisms using molecular dynamics simulations and find that the segmental polymorphic models of Aβ retain a U-shaped architecture. Our results demonstrate the importance of inter-sheet side chain-side chain contacts, hydrophobic contacts among the strands (β1 and β2) and of salt bridges in stabilizing the aggregates. Residues in β-sheet regions have smaller fluctuation while those at the edge and loop region are more mobile. The inter-peptide salt bridges between Asp23 and Lys28 are strong compared to intra-chain salt bridge and there is an exchange of the inter-chain salt-bridge with intra-chain salt bridge. As our results suggest that Aβ exists under physiological conditions as an ensemble of distinct segmental polymorphs, it may be necessary to account in the development of therapeutics for Alzheimer’s disease the differences in structural stability and aggregation behavior of the various Aβ polymorphic forms.

## Introduction

Alzheimer’s disease (AD) is the most common cause of dementia. Implicated in its pathology is the amyloid-β (Aβ) peptide [1], derived from the cleavage of the trans-membrane amyloid precursor protein (APP), which is the main constituent of amyloid plaques associated with the disease. Small soluble oligomers of Aβ peptide are likely the cytotoxic entities [2], [3] that lead to the synaptic dysfunction and cytoskeleton changes underlying the symptoms of Alzheimer’s [4]. Hence, an atomic level understanding of the formation of the amyloid oligomers and protofibrils, and the factors that affect their aggregation, is crucial for the rational design of therapeutic strategies that prevent Aβ aggregation into toxic structures and, perhaps, allow one to treat Alzheimer’s disease.

Amyloid forming proteins aggregate into structurally diverse fibrils due to differences in positioning of polypeptide chains within the fibrils [5]. Recent cryo-electron microscopy studies [6], [7] have shown complex polymorphism of Aβ fibrils characterized by size, cross section and width. These differ from fibrils studied by solid state NMR (ssNMR) [8], [9] in the location of the U-turn as well as the specific interactions between the distal regions, demonstrating that polymorphism is present at the protofilament level [9], [8]. The variety of polymorphs suggests multiple interaction sites within each Aβ molecule giving rise to differences in fibril morphology and variations in the toxicity [10], [11], [12]. Experimental studies have shown that the morphology of Aβ fibrils is highly sensitive to environmental conditions [10], [13]. Polymorphs may also differ in their stability in the amyloid fiber leading to more or fewer infectious seeds, and thus to a difference in infectivity or disease onset rate [11], [14].

Three models for amyloid polymorphs have been proposed on the basis of atomic structures of amyloid-like fibers [15], [16]. The first model is termed packing polymorphism, where an amyloid segment packs into two or more distinct ways, producing fibrils with different structures and distinctive properties [16], [14]. In segmental polymorphism, two or more different segments of an amyloid protein are capable of forming steric-zipper spines [16], [17]. In a third type of amyloid polymorphism, heterosteric zippers are formed from the inter-digitation of non-identical β sheets.

**Figure 1:**
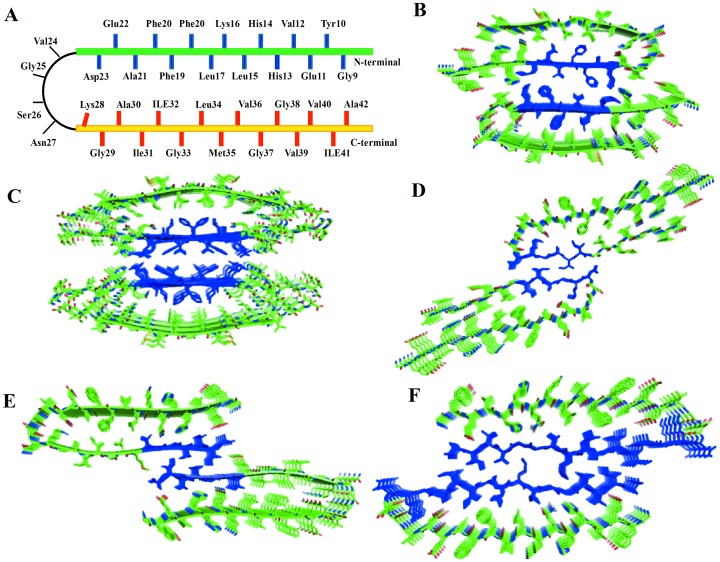
Structural models of double-layer Aβ segmental polymorphism proposed by Eisenberg group. (A) Schematic representation of the U turn structure of Aβ single layer based on ss-NMR. The first beta sheet (green) and the second beta sheet (yellow) are represented by a thick line. The thin (black) line represents the loop region that connects the two sheets. The crystal structure of Aβ_16−21_ form II (blue) serves as an interface for model 16–21P (Figure B) and model 16–21AP (Figure C) of Aβ_16−21_. The model 16–21AP displays antiparallel β sheet. In the model 27–32 (Figure D) interactions between double-layer Aβ is through the crystal structure of Aβ_27−32_. The model 35–42 in Figure 1 E is based on the crystal structures of Aβ_35−42_ form II as the interface between double-layer Aβ. The fifth model (Figure 1F) is based on the long steric zipper interfaces consisting of Aβ_30−35_ and Aβ_35−42_ microcrystal structure. The blue color is used here to indicate the interfacial hydrophobic interactions based on the microcrystal structures.

The distribution of Aβ monomers, the early stages of oligomerization, their dependence on sequence (i.e., mutations) and environment [18], [19], [20], [21], the mechanism of Aβ fibril disassembly [22], [23], [24], [25] and the early steps of Aβ monomer deposition on fibril fragments [26], [27], [28], [29] have been studied extensively *in silico*, using protein coarse-grained lattice [30] and off-lattice models,[31] and all-atom force fields [32]. Nguyen *et al* [33] recently performed a systematic comparison of all atom force fields on the structures and energetic of the monomer, dimer and trimers of Aβ_16−22_. Berryman et al [34] examined the thermodynamic stability of amyloid fibrils in different polymorphic forms, and molecular dynamics on conformational differences in the U-turn of Aβ_17−42_ have indicated that it leads to polymorphism with large differences in energy and populations [35]. However, to our best knowledge, there have not been any numerical studies on the stability of segmental polymorphism of Aβ aggregates. This is the purpose of the present article. Using atomistic molecular dynamics simulations on five different segmental polymorphs models of Aβ with the same U turn but different interface interaction we investigate their stability. All of the five models have residues 23–29 (**Figure 1A**) in the loop region that connects the two β-sheets, composed of residues 10–22 (β1) and 30–40 (β2). Especially, we aim to answer the following questions:

1. Which of the studied segmental polymorphs are more stable in an explicit aqueous system?
2. How does interface size and arrangement into parallel and antiparallel β-sheets influence the stability of different segmental polymorphs?
3. How does a salt bridge between Asp23 and Lys28 stabilize the turn region connecting the two β-strands in the various segmental polymorphs of Aβ?

## Methods

Short segments of amyloid forming proteins can form microcrystal. Their atomic structures consist of a pair of tightly mated β-sheets called steric zippers. The steric zippers form due to inter-digitations of side chains and hold together pair of β-sheets [36]. Colletier *et al*. [37] used eleven microcrystal structures, obtained from various segments of Aβ within the region of residues 16 to 42 and based on previous ssNMR model of Aβ [8], to propose segmental polymorphic models of Aβ. These models exhibit U-shaped, β-strand-turn-β-stand motifs [8], arranged in a parallel manner to maximize the number of hydrophobic contacts and that are further stabilized by the D23-K28 salt bridge.

**Table 1:** Summary of Aβ segmental polymorphic oligomeric models and Simulation Conditions.

Model	Sheet organization	#Peptide/#Water/#Na+	Simulation box (Å)	Interfaces	Time (ns)
16–21P	Parallel/Anti-parallel	4770/25407/30	94.9×94.9×94.9	16–21(NN)	100(50×2)
16–21AP	Anti-Parallel/Anti-parallel	5080/25220/30	92.9×92.9×92.9	16–21(NN)	100(50×2)
27–32	Parallel/Anti-parallel	4770/62511/30	124.6×124.6×124.6	27–32(CC)	100(50×2)
35–42	Parallel/Anti-parallel	5060/53285/30	118.4×118.4×118.4	35–42(CC)	100(50×2)
30–42	Parallel/Anti-parallel	5060/30650/30	99.1×99.1×99.1	30-35-42(CC)	100(50×2)

These atomistic models proposed by the Eisenberg group [37] are shown in the **Figure 1**. The fibril models are constructed from the steric zipper structures of Aβ_35−42_ (**Figure 1E and F**), Aβ_16−21_ (**Figure 1B and C**) and Aβ_27−32_ (**Figure 1D**). The interface between the double layers of the Aβ_16−21_ (**Figure 1B**) is different from the models in **Figure 1D–1F** as the former involves the pairing of N–terminal β-sheets, and in the latter the interface is between C –terminal β-sheets. The second model based on Aβ_16−21_ (**Figure 1C**) steric zipper interface relies on the ss-NMR structure of the D23N Iowa Aβ mutant [38] with its antiparallel β-sheets. The model of the double layer interface that covers residue 30–40 (**Figure 1F**) has the longest interfaces. The double layer models for four of the studied segmental polymorph of Aβ (16–21P, 27–32, 35–42 and 30–42) are based on previously reported Tycko model from ss-NMR studies of Aβ_9−40_ [8].

The simulations are performed with the GROMACS program version 4.5.3 [39] using a time step of 2 fs. We employ the most recent amber force field (ff99SB-ILDN) for the peptide [40] and the TIP3P water model [41] for our simulations. Periodic boundary conditions are employed, and the PME algorithm [42], [43] is used for modeling electrostatic interactions. Atoms involving hydrogens are constrained using the LINCS [44] algorithm (fourth order with one iteration), and for water the Settle algorithm is used [45]. The constant temperature of 330 K is maintained by a temperature coupling with the V-rescale algorithm [46] (τ = 0.1 fs) and pressure coupling with the Parrinello-Rahman algorithm [47] (τ = 1 fs). Energy is minimized by steepest descent followed by conjugate gradient algorithms to remove steric clashes. The simulation is equilibrated in two steps of 500 ps, the first step in an NVT ensemble and the second phase in an NPT ensemble at 1 bar. Each system is simulated for 50 ns at constant pressure (1bar) and the trajectories are saved at 4.0 ps intervals for further analysis. The temperature of 330 K is selected as a compromise between experimental stability of the amyloid fibrils [48] and thermally enhanced sampling [49], [50]. Two independent simulations with different initial velocity distributions are performed for each system to test for thermalization and guarantee at least two independent sets of measurements. A detailed summary of the simulation can be found in Table 1. The coordinates of Aβ segmental polymorphic models were kindly provided by Dr. M. Sawaya [37].

**Figure 2:**
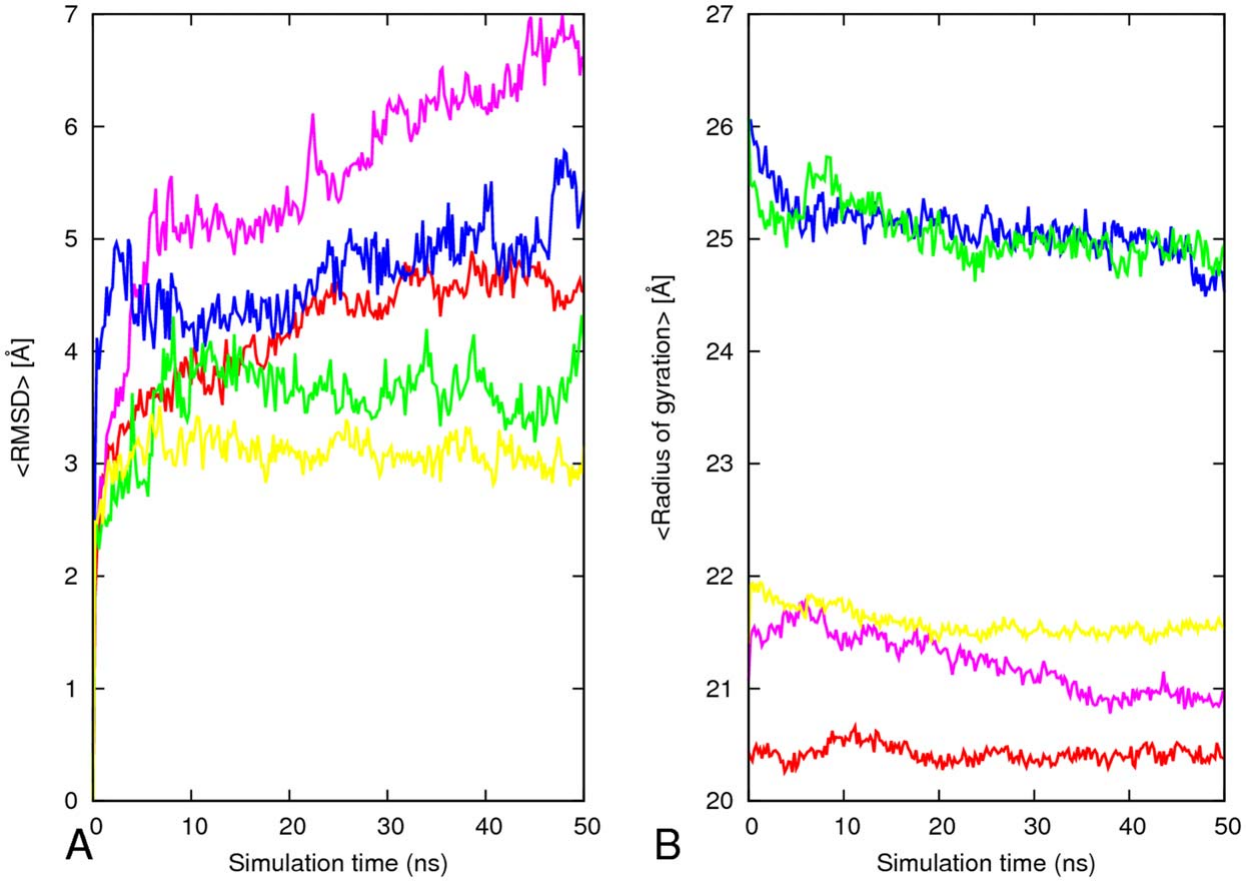
Root Mean Square Deviation (RMSD) and Radius gyration (Rg) for the Aβ segmental polymorphism models. Variation of the C_α_ atom root mean square deviation (RMSD) with respect the energy minimized structure of the five segmental polymorphic models of Aβ. The <RMSD> of each model was calculated using two independent trajectories (A). Radius of gyration as a function of time for each structures during the 50 ns MD simulations (B). Red, 16–21P; pink, 16–21AP; blue, 27–32; green, 35–42; yellow, 30–42.

After equilibration, 50 ns of trajectories are analyzed for each system to examine the structural changes of the oligomers aggregates. We monitor the conformational change and the conservation of the oligomers by the time evolution the root means square deviations of the Cα atoms, radius of gyration, root mean square fluctuations per residue, solvent accessible surface area, inter-strand distances, salt bridge distance variation and secondary structure persistence. We use Visual Molecular Dynamic (VMD) software version 1.9 [51] to display the structural changes of models during the simulation runs.

**Figure 3:**
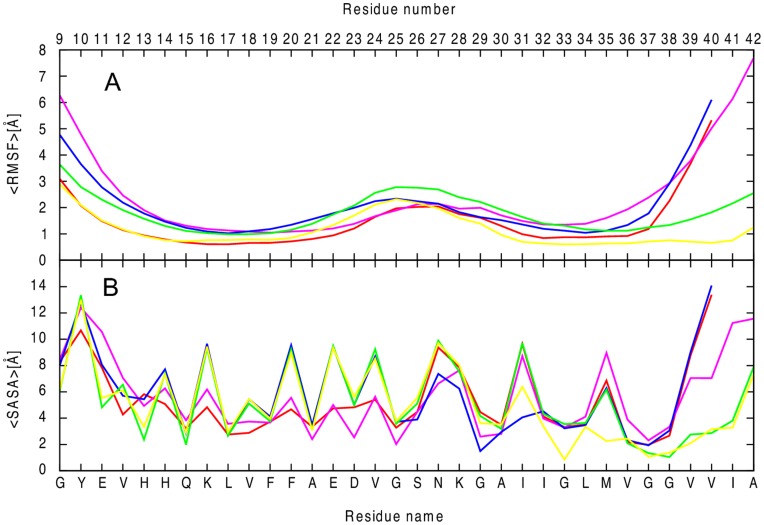
Comparison of all-atom root-mean-square deviation and solvent accessible surface areas of Aβ segmental polymorphism models. Backbone C_α_ atom-positional root-mean-square fluctuations, RMSF, along the amino acid sequence for the five models (A). The results are the average of two independent salutation of each system. The variation of average per residue solvent accessible surface area for each models (B). Red, 16–21P; pink, 16–21AP; blue, 27–32; green, 35–42; yellow, 30–42.

We simulate each model for an additional 20ns simulation in order to calculate the binding free energies in double layer complex and to provide insight into interaction energy and energetic stability of the segmental polymorphs. The simulations are done with the AMBER11 [52] software packages, using the all-atom AMBER99SB [53]. The fibril models are explicitly solvated in a periodic water box of TIP3P molecules, and carefully equilibrated before the production run of 20 ns.

## Results and Discussion

### Conformational Stability of Aβ_9–40/42_ Segmental Polymorphs

We start our analysis by investigating the relative conformational stabilities of the oligomers. These are measured by the root-mean-squared deviation (RMSD) with respect to the initial minimized structure. We find that the backbone RMSDs of the segmental polymorphs of Aβ with the CC interface deviate less than the corresponding oligomers with the NN interface, as shown in **Figure 2A**. The stability of models with CC interface depends on the size of steric zipper and the nature of residue at the interface. Model 27–32 with CC interface stabilized by small size amino acids side chain and few residues at steric zipper interfaces have an average RMSD of about 5 Å, with a reduced stability of its aggregates compared to other models with similar interfaces (Figure 2A). The most stable model among the studied polymorphic models of Aβ is the model 30–42 with longer interface covering residues 30–40. This confirms previous work that probed the stability of the aggregate as function of the size of the steric zipper and the nature of residue [54], [55], [56]. The parallel β-sheet model with NN interface has an average RMSD of 4.2 Å within the last 30 ns as shown in **Figure 2A** (4.2 Å), while the antiparallel (6.2) shows large fluctuations in RMSD within the first 5 ns and then increased to more than 6 Å after 25 ns. This indicates that the parallel structure is more stable than the antiparallel one, which is in agreement with recent experimental results [57], [38].

**Figure 4:**
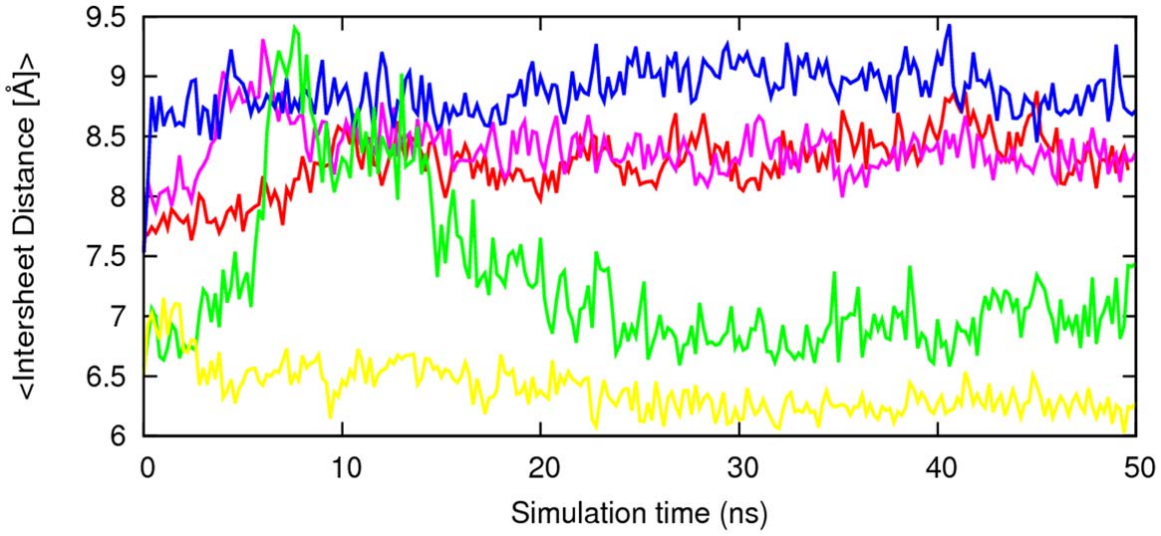
Time evolution of sheet-to-sheet distances. The inter-sheet distances for the models 16–21, 27–32, 30–42 and 35–42 were calculated by averaging the mass center distance between backbone residues of 16–21, 27–32, 30–42 and 35–42 respectively. The results are the average of two independent simulation of each system. Red, 16–21P; green, 16–21AP; blue, 27–32; pink, 35–42; cyano, 30–42.

The radius of gyration is a measure of the mass-weighted spatial distribution of the atoms in a peptide molecule and a rough measure for its compactness. **Figure 2B** shows the radius of gyration of peptide backbone as a function of time. Models with CC interface and smaller steric zipper have the biggest radius of gyration indicating they are elongated while the other three polymorphs have a smaller radius of gyration. In the simulations of 16–21P, 27–32, 35–42 and 30–42 the radius of gyration oscillates near its initial value during most of the simulation. The radius of gyration fluctuates about 0.7 nm for antiparallel model (16–21AP) which also has the largest RMSD values (**Figure 2B**).

**Figure 5:**
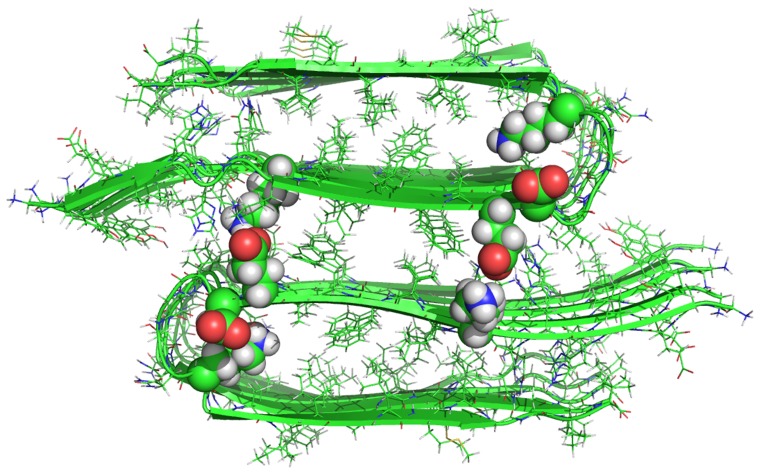
The structure of the starting configuration of of the interactions of Asp^23^/Lys^28^ and Lys^16^/Glu^22^ for the double layer 16–21P model. The positions of the residues originally involved in the formation of the salt bridge are represented in sphere visualization to emphasize their location.

We assess the local dynamics and flexibility of the each part of the five segmental polymorphic models of Aβ by calculating the residue-based root mean square fluctuations (RMSF) of the corresponding backbones with respect to their energy minimized structure. Residues in the turn region exhibited a higher flexibility than those in the β-strand regions, except for residues near the N/C-termini (**Figure 3A**). By visual inspection of the trajectories we find that all 10-mer structures maintain the U turn or “β arch” motif without disassociation of the β-strands. The model based on the 16–21 parallel steric zippers with an NN interface is more stable than the antiparallel counter parts (**Figure 3A**). This is in agreement with the RMSD result above and with the recent ssNMR experimental study of the Iowa mutant of amyloid β [57]. The model covering 30–40 with longer interface is the most stable polymorph, with smaller fluctuation in both β1 and β2. The terminal amino acids of all structures undergo more dynamic reorientation and are more disordered due to exposure to the solvent molecules (**Figure 3A**).

**Figure 6:**
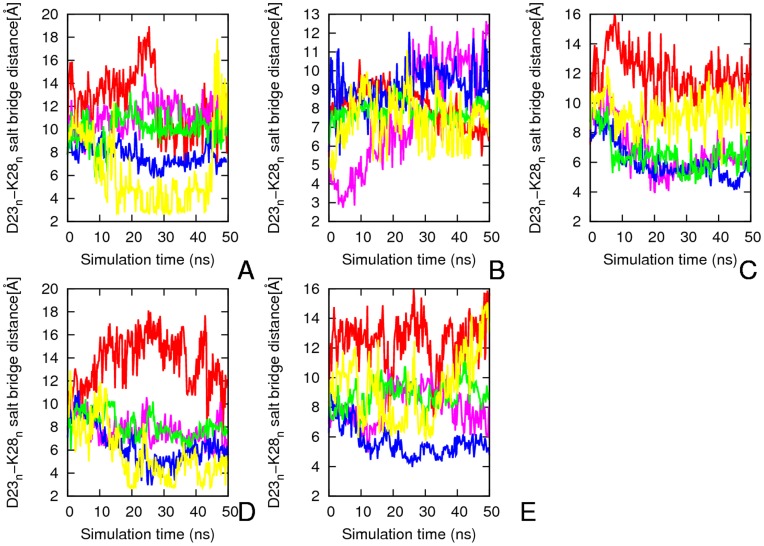
Average intra-chain salt bridge distance (Asp_n_^23^/Lys_n_^23^) along the 50 ns simulation for Aβ segmental polymorphs. The results are the average of two independent simulations and it is the average of the two layers of each system. A) 16–21P B) 16–21AP C) 27–32 D) 35–42 and E) 30–40. Red, _1_D^23^-_1_K^28^; pink, _2_D^23^-_2_K^28^; blue, _3_D^23^-_3_K^28^; green_, 4_D^23^-_4_K^28^; yellow, _5_D^23^-_5_K^28^.

Next we calculate the per residue solvent accessible surface area (SASA) of the various system to investigate its effect on the stability of the models. We compute the SASA using g_sas tool in GROMACS with a probe radius of 1.4 Å, and measure its value for the C-terminal (residues 30–40), N-terminal (residues 10–22), and turn regions (residues 23–29) of each model (**Figure 3B**). The two systems with N-terminal to N-terminal interface (16–21P and 16–21AP) have the edge residues from both the N-terminal and C-terminal β-strands exposed to the bulk solution with a hydrophobic core buried inside (**Figure 3B**). These two double layer models have much large SASA at the edge than at the center indicating that the amino acids at both the N and C-terminal are exposed to the solvent. The charged residues Lys16 and Glu22 are not exposed to the bulk solution but rather form interlayer salt bridges, and thus have a smaller solvent accessible surface area. The 27–32 and 35–42 models with a smaller size C-terminal to C-terminal interface steric zipper have both N-terminal and C-terminal β-strands exposed to the bulk solution, while model 30–42 with its large steric zipper interface exposes only the N-terminal to the solvent, and therefore has relatively small SASA values for hydrophobic C-terminal residues.

**Figure 7:**
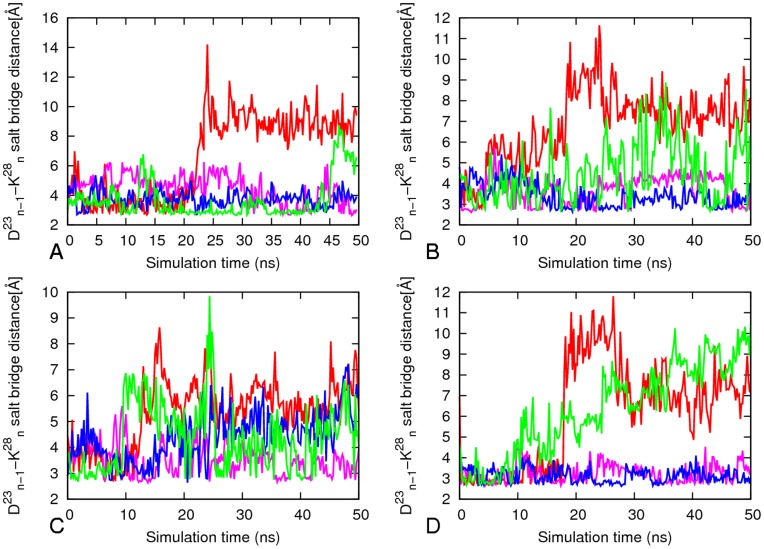
Average inter-chain salt–bridges (Asp_n_^23^/Lys_n−1_^23^) along simulation for 16–21P, 27–32, 30–40 and 35–42. The results are the average of two independent simulations and it is the average of the two layers of each system. A) 1621P B) 27–32 C) 35–42 and D) 30–30. Red, _1_D^23^-_2_K^28^; pink, _2_D^23^-_3_K^28^; blue, _3_D^23^-_4_K^28^; green_, 4_D^23^-_5_K^28^.

The Aβ sequence has two hydrophobic segments (residue 17–21 and 29–40). The five models are stabilized by the interaction between these hydrophobic segments between strands. The models differ in the solvent accessible surface area per residue in the two hydrophobic segments (**Figure 3B**). The hydrophobic segment of residue 17–21 is buried in the 16–21P polymorphs model while in all other models only residues L17 and F19 are buried, resulting in a higher solvent accessible surface area in the later models. In the second hydrophobic segment of Aβ the model 30–42 is protected from solvent almost completely and thus has the smallest solvent accessible area in this region. This explains also the difference in the RMSF of the various polymorphs in the terminal, β-sheet and loop region of the peptides.

We also assess the stability of the sheet-to-sheet associations’ of the double-layered organizations of the models by following the change in the inter-sheet distance across the interface. **Figure 4** shows the averaged distances between the mass centers of two facing β-sheets. The models with NN terminal interfacial associations have an inter-sheet distance of about 8.5 Å. The inter-sheet distance measurement shows larger inter-sheet distance for the NN terminal than CC terminal except for the model 27–35. This is due to the reduced hydrophobic interactions at a NN interface as compared to a CC interface. The segmental polymorphic model 27–32, besides having the smallest size of interface steric zipper, has polar hydrophilic Asn residues at both ends of the interface. The inter-sheet distance for this model increases from the initial 8 Å to 9 Å within the 2 ns of the simulation and remains about 9 Å throughout the remaining simulation time. Its inter-sheet distance measurements shows that the stabilization of the sheet to sheet association is due to good geometrical fit between side chains at the interface leading to a favorable interaction that tighten the packing between *β-*sheets.

**Figure 8:**
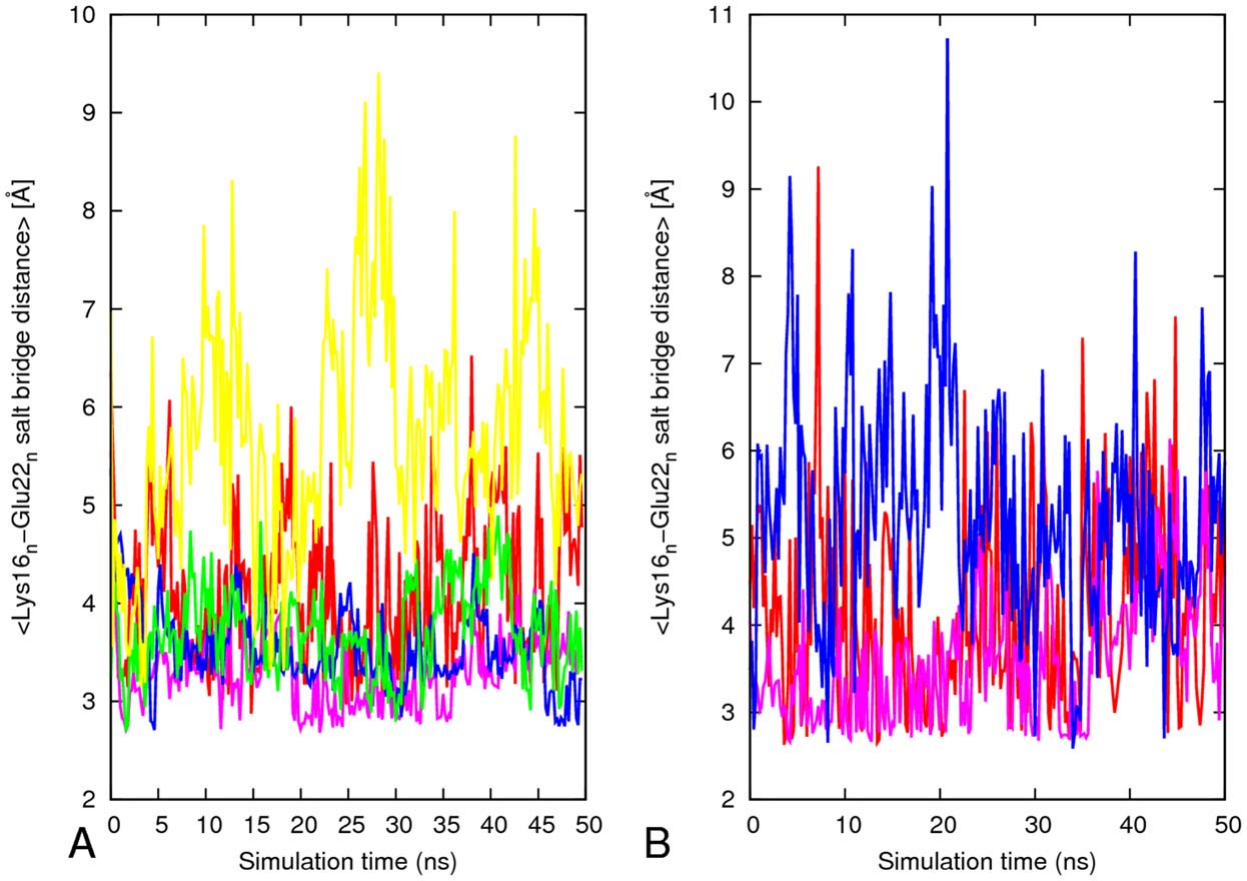
Average inter-sheets salt–bridge distance (Lys_n_^16^/Glu_n_^22^) along simulation for 16–21P and 16–21AP. A) 16–21P and (B) 16–21AP. The results are the average of two independent simulation of each system. Red, _1_K^16^-_1_E^22^; pink, _2_K^16^-_2_E^22^; blue, _3_K^16^-_3_E^22^; green_, 4_K^16^-_4_E^22^; yellow, _5_K^16^-_5_E^22^. Red, 16–21P; green, 16–21AP; blue, 27–32; pink, 35–42; cyano, 30–42.

### Variation of Salt Bridge Distances

The salt bridge between Asp23 and Lys28 [8], [58], [59], has been proposed to stabilize the loop region that connects two β-sheets of the U turn or (β arch) model of Aβ and prevents larger backbone motions. We perform a time dependent analysis of the salt bridge to probe its effect on the stability of the aggregates. The internal Asp23 and Lys28 salt bridge interaction appears in all of the starting conformations of the Aβ segmental polymorph models which is in agreement with pervious experimental [8] and theoretical models of Aβ [59], [35]. In all of the five segmental polymorphs both Asp23 and Lys28 are located in the turn region. The two polymorphic models with N-N terminal interfaces (16–21P, **Figure 1B** and 16–21AP, **Figure 1C**) have an additional salt bridge between Lys16 and Glu22 across the sheet to sheet interface. The salt bridge distance is calculated as the averaged distance of the C = O bonds carboxyl group of Asp 23(or Glu22) to the N atom of the NH_3_^+^ in Lys 28 in the intra-chain salt-bridge (Asp_n_^23^/Lys_n_^23^), inter-chain salt–bridge (Asp_n_^23^/Lys_n−1_^23^) and interlayer salt bridge between Lys16/Glu22 for the models 16–21P and 16–21AP (**Figure 5**). Direct salt bridges are assumed to be around 4.3 Å, whereas indirect or water-mediated salt bridges have a distance between 4.3 and 7.0 Å [60].

**Table 2:** Summary of the MM/PBSA Energy (kcal/mol) Component Analysis of the Bilayer Systems of the MD Simulation of the Double Layer Models of Aβ segmental polymorphism.

Model	<ΔE_ele_>	<ΔE_vdw_>	<ΔG_PB_>	<ΔG_SA_>	<Δ_solv_>	<Δ_binding_>
16–21P	−36.0(2.0)	−334.7(0.4)	118.6(1.7)	−31.2(0.1)	87.3(1.7)	−293.4(0.7)
16–21AP	−369.2(1.3)	−117.6(0.3)	405.9(1.2)	−17.2(0.1)	388.8(1.2)	−98.0(0.4)
27–32	83.7(2.3)	−316.7(0.4)	−44.7(2.1)	−29.4(0.1)	−74.1(2.1)	−307.0(0.4)
35–42	246.4(1.6)	−356.3(0.2)	−135.4(1.5)	−34.7(0.1)	−170.2(1.5)	−280.1(0.4)
30–42	100.3(2.0)	−377.5(0.3)	−28.5(1.7)	−33.3(0.1)	−61.7(1.7)	−338.9(0.6)

ΔE_ele_, nonsolvent electrostatic potential energy; ΔG_PB_, electrostatic contributions to the solvation free energy calculated with Poisson-Boltzmann equation; G_SA_, nonpolar contributions to solvation free energy; ΔEvdw, van der Waals potential energy; ΔG_binding_, calculated binding free energy. Data are shown as mean (Std Err of Mean). ΔG_binding_  =  ΔE_vdw_ + ΔE_ele_ + ΔG_sol_; ΔG_sol_  =  ΔG_PB_ + ΔG_SA._

### Intra-chain Salt-bridge (Asp_n_^23^/Lys_n_^23^)

Almost all of the studied models have a larger intra-chain distance between the Asp23 and Lys28 and most of them do not have a direct salt bridge. The three models with N-N terminal have on an average three (**Figure 6C and D** **)** to one (**Figure 6E**) indirect Asp23-Lys28 salt bridge per layer. The 16–21P model with CC terminal interface forms on average two salt bridges per layer after 10 ns and they were preserved for the most of the MD simulation (**Figure 6A**). The model with antiparallel β–sheet (16–21AP) have an intra-chain salt bridge between Asp23 and Lys28 which is unstable during most of simulation time and is observed only at beginning and the end of simulation (**Figure 6B**).

**Figure 9:**
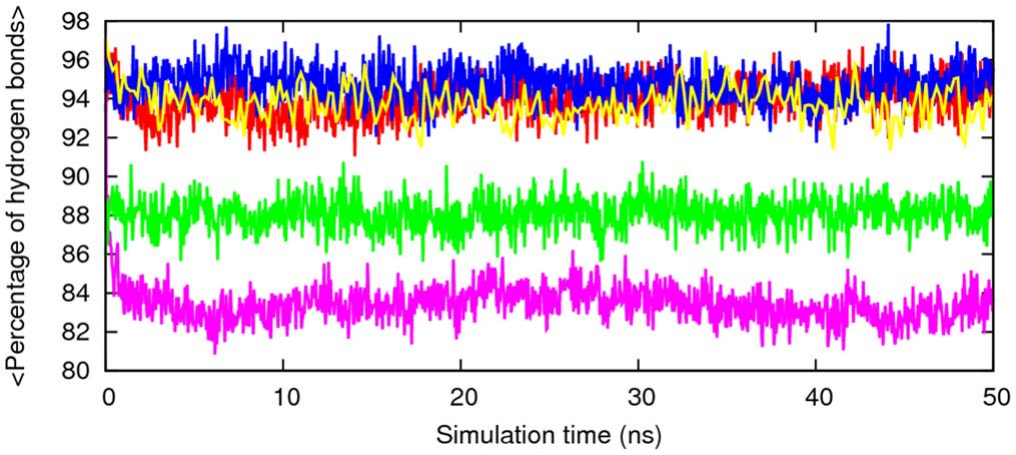
Percentage of hydrogen bonds as a function time with respect to the energy minimized structure of Aβ segmental polymorphic models. Red, 16–21P; green, 16–21AP; blue, 27–32; pink, 35–42; cyano, 30–42.

**Figure 10:**
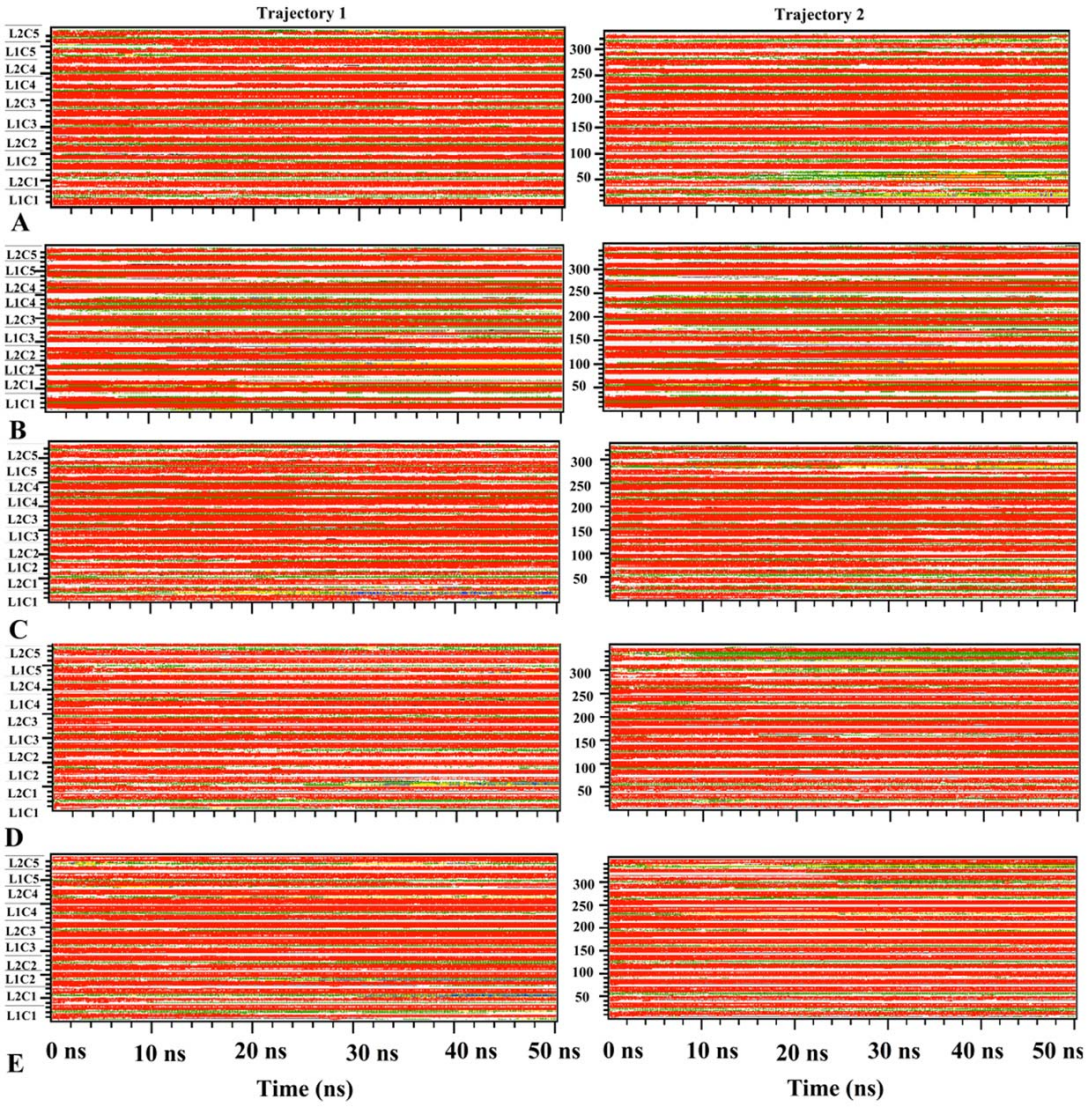
Secondary structure variation plot for each of the Aβ segmental polymorphism models. (A) Aβ_16−21_P, (B) Aβ_16−21_AP (C) Aβ_27−32_, (D) Aβ_35−42_ and (E) Aβ_130−42_ interfaces. The secondary structure color codes: red-β-sheet, green-bend, yellow-turn, blue -α-helix, coil-white. Where L stands for the peptide layers number and C stands for the peptide chain number.

**Figure 11:**
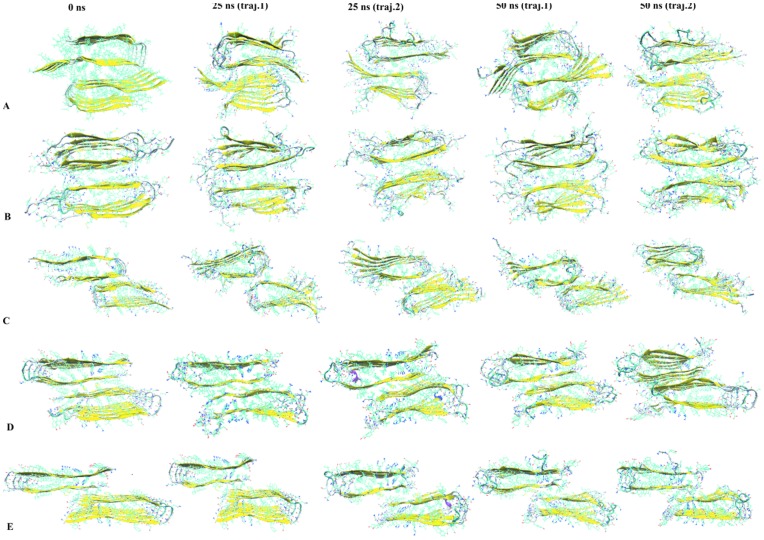
Snapshots from MD simulations for double-layered Aβ segmental polymorphism models with the steric zipper interfaces. (A) Aβ_16−21_P, (B) Aβ_16−21_AP (C) Aβ_27−32_, (D) Aβ_35−42_ and (E) Aβ_130−42_ interfaces at 0ns, 25ns and 50 ns.

### Inter-chain Salt–bridge (Asp_n_^23^/Lys_n−1_^28^)

The direct inter-chain salt bridge between Asp23 and Lys28 is strong (**Figure** **7A**) for model 16–21P, persisting through most of the molecular dynamics simulation. The sole exception is the first salt bridge between _1_D23 and _2_K28 where one of the outer chains is exposed to water and is highly mobile. The inter-chain Asp23-Lys28 salt bridges (with an average of three inter-peptide salt bridges per layer) stabilize the U-shaped conformation and account for its relatively high structural rigidity (**Figure 3A** and **6A**). The models 30–42 and 27–32 have on average two inter-peptide salt bridges while model 35–42 with its largest flexibility in the loop region (**Figure 3A**) has on average only one salt bridge per layer (**Figure 7B–D**). We observe that the aggregates form more stable inter-chain salt bridges than intra-chain salt bridges. This is due to a larger intra chain distance of ∼8.5 Å versus the 3.5 Å inter-chains distance in the initial structure of the aggregates.

### Interlayer Salt Bridge

The charged residues Lys16 and Glu22 are exposed to the bulk of the solution in the models 27–32, 35–32 and 30–42, and all of them lack the Lys16/Glu22 salt bridge. Only the 16–21 models have an inter-sheet Lys16/Glu22 salt bridge. The model 16–21P with parallel β-sheets has a potential to form five salt bridges while the antiparallel can only form three (**Figure 8**). As compared to anti-parallel β-sheets model 16–21AP (RMSD ∼5.0–7 Å, Rg ∼22.5 −21 Å and RMSF ≥ 1.2 Å in the β-sheet region), the parallel β-sheet model 16–21P has only small structural deviation (RMSD ∼3.0–4.5 Å, Rg ∼20.4 Å and RMSF ≤0.9 Å in the β-sheet region). This is due to the enhanced peptide-peptide interactions through salt bridge and hydrophobic interaction in the β-sheet region. Exposure of the outer strands to water leads to high mobility for all external residues, and to the disruption of intra-sheets salt bridge of Lys16/Glu22 (**Figure 8**) at the turn region. This increases the flexibility of the turn in the model 16–21AP which has about one stable interlayer salt bridge compared to three stable interlayer inter-chain salt bridge in the model 16–21P.

### The MM-PBSA Analysis

In order to calculate the binding free energies with the MM-PBSA method, explicit water simulations are used to generate the trajectory followed by the implicit Poisson-Boltzmann/surface area method. The binding free energy is calculated using 5000 snapshots over the course of 20 ns based on the singe-trajectory approach [61]. This approach was previously used to study the thermodynamics of amyloid aggregate stability [56], [49], [62]. MM–PBSA energy contributions are shown in **Table 2**. The MMPBSA calculation of the interaction energies between two β-sheets of the segmental polymorphs indicates that the 16–21P model is more stable than anti-parallel 16–21AP model. The energetically most stable segmental polymorphs in an explicit aqueous system is model 30–42 with longer interface.

The amyloid configuration and properties primarily depend on the density of hydrogen bonds involving the backbone of the polypeptides, while the side chains hydrogen bonds are involved in the geometrical details and extension of the disordered parts of the structure [63], [64]. To further characterize the structural stability of the segmental polymorphism of Aβ models the hydrogen-bonds contents are compared to minimized structure structures. For computing the number of hydrogen bonds, donor-acceptor distance cut-off value assigned is 0.35 nm. The percentage of hydrogen bonds retained through the simulated time with respect to the minimized structure is plotted in **Figure 9** and it indicates the aggregates remain ordered, with less 20% decrease in the original hydrogen bonding.

### Secondary Structure Analysis and Snap Shots of the Structure

Using the dssp tool which determines the existence of hydrogen bonds as criteria for the presence of secondary structure, we analyze the variation of secondary structure during the course of the simulation [65]. The evolution of the secondary structure from two independent trajectories as a function of time is shown in **Figure 10** for each system. The models 16–21P and 30–42 which are found to be more stable aggregates have a higher β –sheet contents than the other three systems (**Figure 10**). Both β-strands of each chain are stable throughout the simulation in all studied systems. However, the peptides located at the ends of the aggregate that occasionally unfold and lose their beta sheet contents. The first two to three amino acid residues in the N terminal and C terminal β-strands adopt a random coil structure throughout the simulations. Snap shots of the segmental polymorphs of Aβ aggregates taken at 0, 25 and 50 ns from two independent trajectories for each of the systems are shown in **Figure 11**. Visual inspection indicates that the U shaped architecture is retained in most of the system. Residues at N terminal and C terminal and loop region show higher mobility in all models. The inner strands have greater structural stability compared to outer strands that are structurally more flexible (see **Figure 11**). The outer peptide chains, despite being unstable, do not dissociate from the aggregates. Hence, our analysis of the time evolution of the proposed segmental polymorphs of Aβ indicates that all models are stable and retain the overall U turn structure.

Pervious molecular dynamics studies on the stability of Aβ aggregates have shown that the two β–sheet regions along with the intervening loop regions exhibit relative rigid and well ordered structure compared to the terminal regions. The loop region which connects the two sheets is stabilized by a salt bridge between the Asp23 and Lys28 that stabilize the short loop connection and prevent large backbone motion. These studies also indicate that both intra and inter-chain D23-K28 salt bridge are maintained during the simulation. We observe a similar picture for the Aβ segmental polymorphic models. The salt bridges in our simulation form the intra-chain and inter-chain salt bridge. The inter-chain salt bridges are more stable than the intra-chain salt bridge. This is due to the larger the intra-chain distance (∼8.5 Å) between the carboxyl group of Asp23 and amine group of Lys28 compared to the ssNMR models in which the distance is much shorter. Visual inspection of the trajectories from all our simulation shows that in the turn region a narrow water channel solvates the interior of the D23-K28 salt bridge as has been reported also in pervious simulation studies of Aβ aggregates [66], [59].

Numerous MD studies have examined the stability of wild type and mutants of Aβ aggregates [59], [12], [67] and its U-turn polymorphism [35] but there have not been previously any numerical studies on the stability of segmental polymorphism of Aβ aggregates, the focus of the present work. Our molecular dynamics simulations indicate that the inter-sheet side chain-side chain interaction, hydrophobic interaction among the strands (β1 and β2) and salt bridge are important in stabilizing the aggregates. We find that

1. The segmental polymorphs of Aβ with the CC interface deviate less than the corresponding oligomers with the NN interface.
2. The stability of models with CC interface depends on the size of steric zipper and the nature of residue at the interface. The segmental polymorph with smaller size of steric zipper shows a larger structural fluctuation while the one with larger size of steric zipper at the interface is very stable. The double layer Aβ based on microcrystal steric zipper interfaces of 16–21 with antiparallel β-sheet organization is found to be unstable than the model with parallel β–sheet. Despite some difference in their structural stability the segmental polymorphic models of Aβ keep their U-shaped architecture with only small fluctuations in β-sheet region. Residues at the edge and loop region show higher mobility.
3. The inter-peptide salt bridges between Asp23 and Lys28 are strong compared to intra-chain salt bridge and there is an exchange of the inter-chain salt-bridge with intra-chain salt bridge.

The knowledge of structural stability and aggregation behavior of Aβ segmental polymorphic may help to develop therapeutics for Alzheimer’s disease. A recent study has shown different aggregation inhibitor molecules bind to different polymorphs of amyloid peptides [68]. Our simulation indicates that a variety of segmental polymorphs can exist at physiological conditions. This suggests that it could be necessary to use as a template for Aβ aggregation inhibitor design not one but multiple microcrystal segments at the double layer interface.
